# A new integrative weaning index of discontinuation from mechanical ventilation

**DOI:** 10.1186/cc8051

**Published:** 2009-09-22

**Authors:** Sergio N Nemer, Carmen SV Barbas, Jefferson B Caldeira, Thiago C Cárias, Ricardo G Santos, Luiz C Almeida, Leandro M Azeredo, Rosângela A Noé, Bruno S Guimarães, Paulo C Souza

**Affiliations:** 1 Critical Care Department, Hospital de Clínicas de Niterói, Rua La Salle 12, Centro, Niterói, Rio de Janeiro, CEP: 24020-090, Brazil; 2 Pulmonary and Critical Care Department, Hospital das Clínicas de São Paulo, Avenida Dr. Enéas de Carvalho Aguiar, sétimo andar, sala 7079, São Paulo, CEP: 05403-000, Brazil; 3 Biostatistics Department, Universidade Federal do Rio de Janeiro, Gávea, Rio de Janeiro, 22631-180, Brazil

## Abstract

**Introduction:**

Indexes predicting weaning outcome are frequently inaccurate. We developed a new integrative weaning index aimed at improving the accuracy of the traditional indexes.

**Methods:**

Three hundred and thirty-one patients mechanically-ventilated for more than 24 hours were evaluated. Initially, the threshold values of each index that best discriminate between a successful and an unsuccessful weaning outcome were determined in 115 patients. In the second phase, the predictive performance of these values was tested prospectively in the other 216 patients. Frequency/tidal volume ratio (f/Vt ratio), tidal volume (Vt), tracheal airway occlusion pressure 0.1 s (P 0.1), the product of P 0.1 and f/Vt (P 0.1 × f/Vt), respiratory rate (f), static compliance of the respiratory system (Cst,rs), ratio of arterial oxygen tension to fraction of inspired oxygen (PaO_2_/FiO_2 _ratio) and the new integrative weaning index IWI (Cst,rs × arterial oxygen saturation/f/Vt ratio) were evaluated in all patients. The readiness for weaning and the decision to return to mechanical ventilation was made by the physician in charge, based on the signs of poor tolerance. The receiver operating characteristic (ROC) curves were calculated in order to evaluate the predictive performance of each index. The Bayes' theorem was used to assess the probability of each test of predicting weaning.

**Results:**

In the prospective-validation set, successful weaning was observed in 183 patients (84.7%) and weaning failure in 33 (15.27%). IWI presented the highest accuracy, with the area under the ROC curves larger than that under the curves for the f/Vt ratio (0.96 × 0.85 respectively; *P *= 0.003), and also larger than that under the curves for the other indexes. IWI presented a higher probability of successful weaning when the test was positive (0.99) and a lower probability when the test was negative (0.14). Measurement of Cst,rs during the weaning process was considered one of the study limitations.

**Conclusions:**

IWI was the best predictive performance index of weaning outcome and can be used in the intensive care unit setting.

**Trial Registration:**

controlled-trials.com ISRCTN92117906

## Introduction

To date, no weaning predictive index has proven to be ideal [1]. According to the Sixth International Consensus Conference on Intensive Care Medicine [2], patients who meet the following satisfactory criteria should be considered ready for weaning: frequency to tidal volume ratio (f/Vt) less than 105 breaths/min/L, respiratory rate (f) of 35 breaths/min or less, maximal inspiratory pressure (MIP) of -20 or less to -25 cmH_2_O, tidal volume (Vt) more than 5 mL/kg, vital capacity more than 10 mL/kg, and arterial oxygen saturation (SaO_2_) above 90% with a fraction of inspired oxygen (FiO_2_) of 0.4 or less (or partial pressure of arterial oxygen (PaO_2_)/FiO_2 _ratio of 150 mmHg or above). After the assessment of these indexes regarding readiness for weaning, a spontaneous breathing trial (SBT) should follow as a diagnostic test to determine the likelihood of successful extubation [3].

Weaning decisions based only on expert clinical judgment are not always correct [4,5]. Premature discontinuation places severe stress on the respiratory and cardiovascular systems [4], while unnecessary delays can lead to diaphragmatic atrophy [6] that can worsen its force generation and, as a consequence, the MIP. Several predictors of weaning are therefore used to aid decision-making [2].

On reviewing the evidence base for ventilator weaning [7], none of the predictors of weaning demonstrate more than modest accuracy in predicting the weaning outcome. In the McMaster review and guidelines [8,9], 66 predictors of weaning were reviewed and analyzed. Only eight, including the rapid shallow breathing index (RSBI) or the f/Vt ratio, presented significant likelihood ratios to predict the weaning outcome [8,9]. The f/Vt ratio was evaluated by at least 22 studies [10,11], and can be considered the most used predictor of weaning.

The daily screening of the respiratory function followed by SBTs in selected patients can reduce the time of mechanical ventilation and the cost of intensive care, besides being associated with fewer complications [5]. As many factors can be responsible for weaning failure, we hypothesized that a weaning index that integrates significant physiological weaning parameters could be a better index predictor than the traditional ones [11].

The objective of this study is to test the predictive performance of a new integrative weaning index (IWI). We evaluated two groups of patients. In the first one (training set), the threshold values for each weaning parameter were selected. In the second group, we tested the predictive performance of the selected values in a prospective-validation data set of patients.

## Materials and methods

Three hundred and thirty-one patients who were on mechanical ventilation for more than 24 hours were evaluated. Patients younger than 18 years of age or with neurological and neuromuscular diseases were excluded from the study. The study was conducted from September 2004 to January 2008 in three general intensive care units (ICUs) of the Hospital de Clínicas de Niterói (Rio de Janeiro - Brazil), totaling 27 beds. It was approved by the ethics committees of our institution and registered as an International Standard Randomized Controlled Trial under number 92117906. Informed consent was obtained from each patient, whenever possible, or from the patient's next of kin. The ventilators used were Evita 2 (Dräger, Lübeck, Germany). Discontinuation from mechanical ventilation was attempted when the physician in charge judged that the patient was ready to be weaned, according to the following criteria: the cause for starting mechanical ventilation had resolved or at least improved; body temperature was below 38.5°C; hemoglobin was equal to or higher than 8 g/dl; and none or a minimal dose of vasoactive or sedative drugs was administered. A PaO_2 _of 60 mm Hg or more or SaO_2 _of 90% or more with a FiO_2 _of 0.4 or less and positive end-expiratory pressure (PEEP) of 8 cmH_2_O or less were other criteria to be met. A SBT was then evaluated by means of a 2-hour T-piece.

The static compliance of the respiratory system (Cst,rs) was measured in volume control ventilation through the same method used by Aboussouan and colleagues [12], after assessing the digital display of the ventilator to verify the pressure-time curve without inspiratory efforts of the patients. An inspiratory hold for 0.5 to 1.0 second was used to measure the compliance. Cst,rs was calculated by dividing the Vt by the difference between inspiratory plateau pressure and PEEP. A bedside spirometer (Ohmeda RM 121, Tokyo, Japan) was attached to the expiratory valve of the ventilator in order to check the Vt measurement before each calculation of Cst,rs.

Before the weaning trial, all patients were being ventilated in pressure support ventilation 8 to 10 cmH_2_O and PEEP 5 cmH_2_O. To measure tracheal airway occlusion pressure (P 0.1), pressure support was reduced to 7 cmH_2_O and the P 0.1 value was obtained from the average of three consecutive measurements with intervals of 15 seconds [13,14]. A sample of arterial blood to analyze the SaO_2 _and the PaO_2_/FiO_2 _ratio was collected in pressure support 7 cmH_2_O, PEEP 5 cmH_2_O and FiO_2 _0.35.

During the first minute after discontinuation from mechanical ventilation, spontaneous minute volume and respiratory rate were measured with a bedside spirometer (Ohmeda RM 121, Tokyo, Japan) attached to the airway. The spontaneous Vt was calculated by dividing minute volume by f, and the f/Vt ratio was calculated by dividing f by Vt (in liters) [11]. The indexes were measured by the respiratory physiotherapists before the SBTs. The decision to return to mechanical ventilation was made by the physician in charge (who was completely blind to the study and the results of the indexes evaluated), based on the signs of poor tolerance incorporated in our daily routine.

Weaning was considered successful if spontaneous breathing was sustained for more than 48 hours after extubation [2]. During the two-hour period of SBT, tolerance was continuously evaluated by the physician in charge. When the patient remained stable after the two-hour period of SBT, the endotracheal tube was removed. The trial was stopped when at least one of the following poor tolerance criteria was present: SaO_2 _less than 90% and PaO_2 _less than 60 mmHg with FiO_2 _less than 0.5 or SaO_2 _less than 88% and PaO_2 _less than 55 mmHg with FiO_2 _less than 0.5 in patients with chronic obstructive pulmonary disease (COPD); partial pressure of arterial carbon dioxide (PaCO_2_) more than 50 mmHg (or increased by 8 mmHg or more in COPD patients); arterial pH of 7.33 or less or decreased by 0.07 or more; f more than 38 breaths per minute or increased by 50% for five minutes or longer; heart rate of more than 140 beats per minute or a sustained increase or decrease in more than 20%; systolic blood pressure of more than 180 mmHg or less than 90 mmHg; or in the presence of agitation, diaphoresis, disorientation or depressed mental status. A clearly audible cough and adequate mental status were requirements for patients to be considered ready for extubation [15].

Weaning failure was determined if one of the following criteria occurred: failed SBT; reintubation and/or resumption of ventilatory support within 48 hours following successful extubation; or death within 48 hours following extubation [2]. The distinction between weaning failure (inability to tolerate spontaneous breathing without ventilatory support) and extubation failure (inability to tolerate removal of translaryngeal tube) was taken into account [15], although for results and statistical analysis considerations, all extubation failure patients were also regarded as weaning failure.

### The integrative weaning index

The IWI uses three essential parameters that lend themselves to easy measurement and are independent of the patient's cooperation. The IWI evaluates, in a single equation, the respiratory mechanics, the oxygenation, and the respiratory pattern, through Cst,rs, SaO_2 _and f/Vt ratio respectively.

Several reasons concurred to the choice of the parameters above: f/Vt is considered the best [4] or one of the best indexes [8,16] to evaluate the weaning outcome; Cst,rs is associated with a shorter time to weaning when more than 20 ml/cmH_2_O [12]; and SaO_2 _has proven to be useful to evaluate the readiness for weaning or to indicate the weaning failure in several studies and revisions [1-3,5]. Multiplying the respiratory compliance by SaO_2_, we can detect those patients who can or cannot maintain a good oxygenation, despite good or bad respiratory mechanics. Dividing this product by the f/Vt ratio, we can detect those patients who will or will not be able to maintain unassisted breathing. Cst,rs and SaO_2 _are generally directly proportional and inversely proportional to f/Vt ratio, as Cst,rs and SaO_2 _gets higher, f/Vt possibly gets lower. The higher Cst,rs and SaO_2_, the lower f/Vt ratio and IWI tends to be higher.

In order to evaluate the predictive performance of IWI, we compared it with the f/Vt ratio, which has been shown to be the most accurate predictor of failure and success in weaning from mechanical ventilation in the study by Yang and Tobin [11].

We also compared IWI with the following parameters: PaO_2_/FiO_2 _ratio, which represents an important index to evaluate oxygenation; tracheal P 0.1, which is an estimate of neuromuscular drive and is considered an important indicator of successful weaning, mainly in patients with chronic obstructive pulmonary disease (COPD) [13]; and the product of tracheal P 0.1 and f/Vt ratio (P 0.1 × f/Vt), which has shown more specificity compared with its components [14,17], f, Vt and Cst,rs.

The study was divided into two sets: the first set was derived from the data concerning 115 patients. In this phase, data were used to select the cut-off value for weaning parameters. The selected values were those that resulted in the fewest false classifications. The second set was derived from the data concerning the other 216 patients.

### Statistical analysis

Continuous variables were presented as mean and standard deviation, categorical variables as frequencies and percentages. Student's *t *test was used to compare parametric variables and Mann-Whitney test to compare non-parametric ones.

*P *values less than 0.05 were considered significant. The statistical analysis was performed using SAS software (SAS software package, version 9.0; SAS Institute Inc, Cary, NC, USA).

Sensitivity (SE = true positive/true positive + false negative), specificity (SP = true negative/true negative + false positive), positive predictive value (PPV = true positive/true positive + false positive), negative predictive value (NPV = true negative/true negative + false negative) and diagnostic accuracy (DA) = (true positive + true negative)/(true positive + true negative + false positive + false negative) were used to evaluate each index.

The predictive performance of each index was also evaluated by calculating the area under the receiver operator characteristic (ROC) curves [11,18]. The area under the ROC curves for each index was calculated by the nonparametric method of Hanley and McNeil [19] and compared through a technique developed by the same authors [19]. Classified according to the guideline proposed by Swets [20]: area under the curve of 0.5 is a non-informative result; area under the curve of more than 0.5 and 0.7 or less is less accurate; area under the curve of more than 0.7 and 0.9 or less is moderately accurate; area under the curve of more than 0.9 and less than 1 is highly accurate; and area under the curve of 1 is a perfect test.

In the prospective validation set, the prevalence of weaning success and weaning failure was calculated. The likelihood ratio of a positive test (LR+) and the likelihood ratio of a negative test (LR-) were calculated for each index. Likelihood ratios between 0.5 and 2.0 indicate that the weaning parameter is associated with small changes in the post-test probability of success or failure. Likelihood ratios from 2 to 5 and from 0.3 to 0.5 correlate with small but potentially important changes in probability, while ratios from 5 to 10 or 0.1 to 0.3 correlate with more clinically important changes in probability. Ratios higher than 10 or lower than 0.1 correlate with very large changes in probability [21].

We used Bayes' theorem to assess the performance of each test in predicting weaning outcome as a function of the prevalence of weaning success or failure in the prospective validation-set [14]. Bayes' theorem allows the calculation of success or failure of weaning after the performance of a test (post-test probability) [21].

## Results

Three hundred and thirty-one patients were evaluated, 115 in a training set and 216 in a prospective-validation set. In the training set and prospective-validation set, successful weaning was observed in 94 (81.7%) and 183 (84.7%) patients, respectively. In the training set, 17 (81%) of the 21 weaning failure patients did not tolerate the SBT, while 4 (19%) completed the SBT, but required reintubation within the following 48 hours after extubation (extubation failure). In the prospective-validation set, 27 (82%) of the 33 weaning failure patients did not tolerate the SBT, while 6 (18%) completed the SBT, but required reintubation within the following 48 hours after extubation (extubation failure). In the total population, weaning failure was observed in 54 of 331 patients (16.35%, including 10 reintubated patients, 4 of whom died).

Clinical characteristics of the patients in the training set, prospective-validation data set and total population are shown in Table 1. In the prospective-validation set, the prevalence of weaning success was 0.85 (183/216), and weaning failure was 0.15 (33/216). In the entire study, the prevalence of weaning success was 0.83 (277/331), and weaning failure was 0.16 (54/331).

**Table 1:** Clinical characteristics, incidence of successful weaning and weaning failure, and the cause of acute respiratory failure in the training set, prospective-validation data set and in the total population

Clinical characteristics	Training set (n = 115)	Prospective-validation set (n = 216)	Total population (n = 331)
Age (years)	64.68 ± 16.77	63.95 ± 17.63	64.20 ± 17.32
Duration of MV (days)	9.80 ± 8.47	8.78 ± 7.17	9.14 ± 7.65
APACHE II	15.99 ± 5.80	16.02 ± 5.43	16.01 ± 5.55
Successful weaning, n (%)	94 (81.7)	183 (84.7)	277 (83.68)
Weaning failure, n (%)	21 (18.3)	33 (15.3)	54 (16.31)
**Cause of ARF**			
COPD, n (%)	33 (28.69)	65 (30.09)	98 (29.6)
Pneumonia, n (%)	25 (21.73)	43 (19.9)	68 (20.54)
Postoperative ARF, n (%)	23 (20)	40 (18.51)	63 (19.03)
Sepsis, n (%)	12 (10.43)	27 (12.5)	39 (11.78)
ARDS/ALI, n (%)	8 (6.95)	17 (7.87)	25 (7.55)
Miscellaneous, n (%)	6 (5.21)	11 (5.09)	17 (5.13)
Multiple trauma, without brain injury, n (%)	4 (3.47)	7 (3.24)	11 (3.32)
Acute pulmonary edema, n (%)	4 (3.47)	6 (2.77)	10 (3.02)

ALI = acute lung injury; APACHE II = Acute Physiology and Chronic Health Evaluation; ARDS = cute respiratory distress syndrome; ARF = acute respiratory failure; COPD = chronic obstructive pulmonary disease; MV = mechanical ventilation.

In training set, the threshold values of each index that best discriminate between successful or unsuccessful weaning were: PaO_2_/FiO_2 _ratio of 255 or more; Cst,rs of 30 ml/cmH_2_O or more; IWI of 25 ml/cmH_2_O breaths/minute/liter or more; P 0.1 of 3.1 cmH_2_O or less; f of 30 breaths/minute or less; Vt of 315 ml or more; f/Vt ratio of 100 breaths/minute/liter or less; and P 0.1 × f/Vt ratio of 270 cm H_2_O/min/liter or less.

The accuracy, likelihood ratio, probability of weaning success when test is positive and probability of weaning success when test is negative of the indexes utilized to predict the weaning outcome in the prospective-validation data set are shown in Table 2. IWI presented the highest SE (0.97), SP (0.94), PPV (0.99), NPV (0.86), DA (0.97) and likelihood ratio of positive test (16.05) besides the lowest likelihood ratio of negative test (0.03). Moreover, IWI presented the highest probability of weaning success when the test is positive (0.99) and the lowest probability of weaning success when the test is negative (0.14).

**Table 2:** Accuracy, likelihood ratio, probability for weaning success when test is positive and probability for weaning success when test is negative of the indexes used to predict the weaning outcome in the prospective-validation data set

Index	Sensitivity (%)	Specificity (%)	Positive predictive value (%)	Negative predictive value (%)	Diagnostic accuracy (%)	LR+	LR-	P(W+/T+)	P(W+/T-)
PaO_2_/FiO_2_	0.60	0.67	0.91	0.23	0.61	1.79	0.61	0.91	0.77

Cst,rs	0.82	0.76	0.95	0.44	0.81	3.40	0.23	0.95	0.56

IWI	0.97	0.94	0.99	0.86	0.97	16.05	0.03	0.99	0.14

P 0.1	0.76	0.70	0.93	0.35	0.75	2.52	0.34	0.93	0.65

f	0.79	0.58	0.91	0.33	0.76	1.87	0.36	0.91	0.67

Vt	0.76	0.73	0.94	0.36	0.76	2.81	0.32	0.94	0.64

f/Vt × P 0.1	0.76	0.73	0.94	0.36	0.76	2.81	0.32	0.94	0.64

f/Vt	0.81	0.73	0.94	0.41	0.80	2.99	0.26	0.94	0.59

Cst,rs = static compliance of the respiratory system; f = respiratory rate; f/Vt ratio = frequency to tidal volume ratio; IWI = integrative weaning index; LR+ = likelihood ratio of positive test; LR- = likelihood ratio of negative test; P 0.1 = airway occlusion pressure; PaO_2_/FiO_2 _ratio = ratio of arterial oxygen tension to fraction of inspired oxygen; P(W+/T+) = probability for weaning success if test is positive; P(W+/T-) = probability for weaning success if test is negative; Vt = tidal volume.

**Figure 1:**
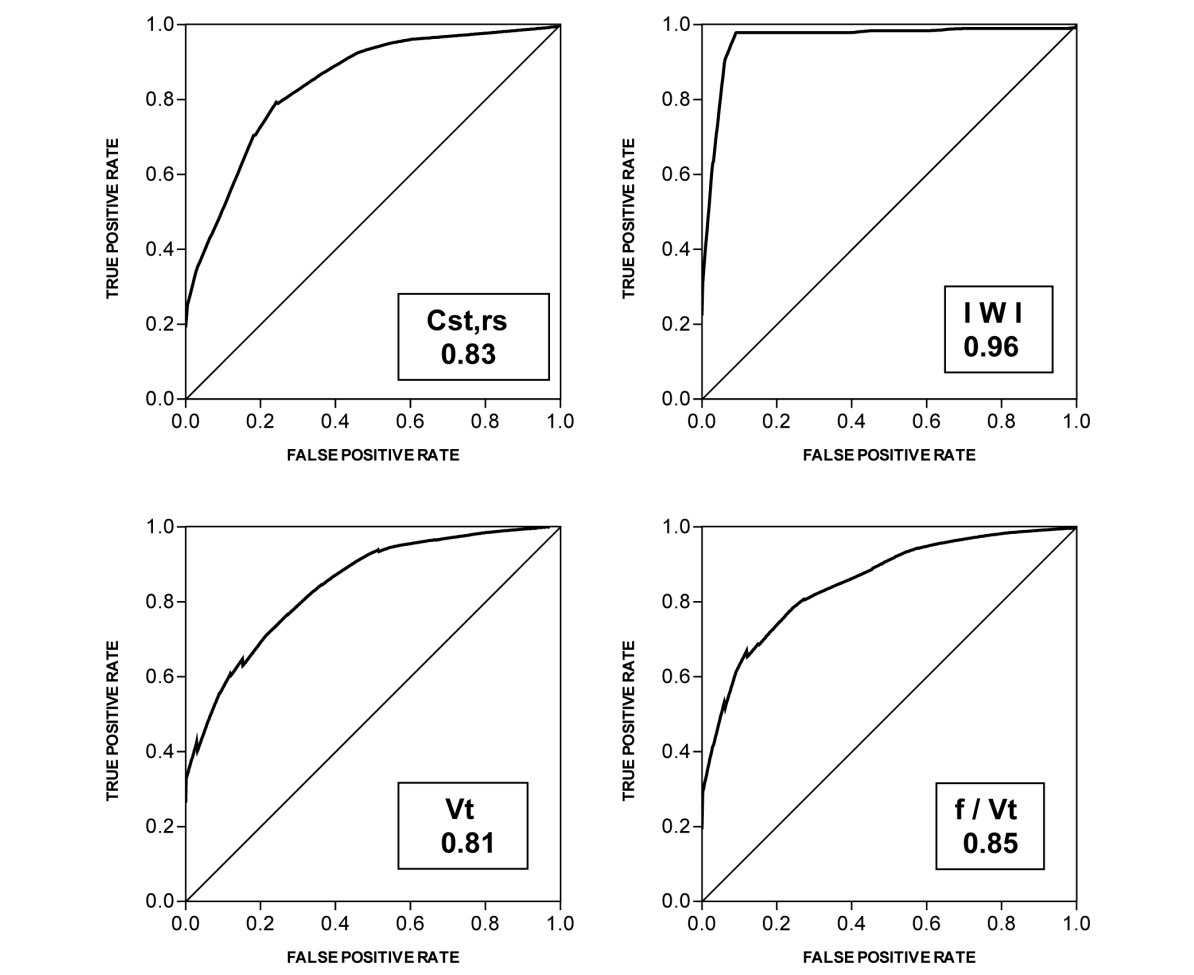
Receiver operator characteristic curves for the indexes evaluated in the prospective validation data set.  f/Vt ratio = frequency to tidal volume ratio; IWI = integrative weaning index; Vt = tidal volume.

The area under the ROC curves for IWI was significantly higher than the corresponding area for the f/Vt ratio (0.96 ± 0.02 × 0.85 ± 0.04 respectively; *P *= 0.003) and also significantly higher than the other indexes. The area under the ROC curves for all the indexes are shown in Table 3 and the comparisons among the area under the ROC curves for all the indexes in the prospective-validation data set are shown in Table 4. Selected most significant ROC curves, that is, for IWI, f/Vt ratio, Cst,rs and Vt, are shown in Figure 1.

**Table 3:** Area under the receiver operator characteristic curves and standard error for each index in the prospective-validation data set

Index	Area + SE
**Cst,rs**	0.83 ± 0.04
**IWI**	0.96 ± 0.02
**P 0.1**	0.72 ± 0.05
**f**	0.73 ± 0.05
**Vt**	0.81 + 0.05
**f/Vt × P 0.1**	0.80 + 0.05
**f/Vt**	0.85 + 0.04
**PaO_**2**_/FiO_**2**_**	0.65 ± 0.06

f = respiratory rate; f/Vt ratio = frequency to tidal volume ratio; IWI = integrative weaning index; P 0.1 = airway occlusion pressure; PaO_2_/FiO_2 _ratio = ratio of arterial oxygen tension to fraction of inspired oxygen; SE = standard error; Vt = tidal volume.

**Table 4:** Comparison of the areas under the receiver operator characteristic curves (*P *value for the two-tailed test)

Index	**PaO_**2**_**/**FiO**_**2**_	Cst,rs	IWI	P 0.1	f	Vt	f/Vt × P 0.1
**Cst,rs**	0.01						
**IWI**	<0.0001	0.002					
**P 0.1**	0.38	0.09	0.00001				
**f**	0.29	0.12	<0.00001	0.88			
**Vt**	0.024	0.75	0.0006	0.15	0.2		
**f/Vt × P 0.1**	0.037	0.63	0.0006	0.021	0.11	0.84	
**f/Vt**	0.003	0.77	0.003	0.005	0.004	0.30	0.20

f = respiratory rate; f/Vt ratio = frequency to tidal volume ratio; IWI = integrative weaning index; P 0.1 = airway occlusion pressure; PaO_2_/FiO_2 _ratio = ratio of arterial oxygen tension to fraction of inspired oxygen; Vt = tidal volume.

## Discussion

The purpose of weaning indexes is to identify patients who can be successfully weaned. Clinical judgment is not enough to predict weaning outcome accurately [5,8] (50% PPV and 67% NPV) [5,22]. The search for better indexes or parameters that can best predict weaning outcome has been attempted by most international weaning researchers. SBT were introduced lately showing a positive weaning predictive value of 85% [5]. However, 15% of the patients who can complete an SBT require reintubation in the following 48 hours after extubation. This indicates that there are patients that tolerate short SBTs but not longer ones. Although SBT represented an advancement, it is not totally satisfying. In the study by Frutos-Vivar and colleagues [23], extubation failure occurred in 121 of the 900 patients (13.4%) that completed the SBT. Among the routinely measured clinical variables, f/Vt ratio, positive fluid balance 24 hours prior to extubation, and the presence of pneumonia at the beginning of mechanical ventilation were the best predictors of extubation failure [23]. This fact reinforces the hypothesis that not only the clinical evaluation, but also the evaluation of weaning indexes (as the f/Vt ratio) could be helpful.

In our study, 18% of the patients that completed the SBT were reintubated. Interestingly, our new index IWI predicted extubation failure in 9 out of 10 patients that presented extubation failure. Our results showed that IWI was useful to detect those patients who passed the SBT but needed reintubation afterwards. Further studies are needed to better understand why IWI can detect this population that fails SBT in a late phase. The IWI presented the highest probability of weaning success when the test was positive (0.99) and the lowest probability of weaning success when the test was negative (0.14). The likelihood ratios of positive test and negative test of the IWI were 16.05 and 0.03 respectively, being correlated with great changes in probability [21]. The area under the ROC curves for IWI was significantly higher than the area of f/Vt ratio (0.96 ± 0.02 × 0.85 ± 0.04 respectively; *P *= 0.003), and also significantly higher than the other indexes, being considered highly accurate, according to the guideline proposed by Swets [20].

Some physiological measurements such as MIP, which generally present the area under the ROC curves considered less accurate [11,21], can be helpful when their values are more than -15 to -10 cmH_2_O, indicating that the patient probably will not be able to breathe spontaneously for a long time. Weaning indexes that evaluate one single function have generally presented poor accuracy [8,11]. For this reason, an integrative index that can evaluate multiple essential functions, such as f/Vt ratio and the compliance, rate, oxygenation and pressure (CROP) index [11] have been introduced in the literature. Unfortunately, the CROP index did not present the same accuracy as f/Vt ratio and its area under the ROC curves was no more than 0.78 [11]. In our study, the new integrative IWI presented the area under the ROC curve of 0.96 and f/Vt of 0.85. Our hypothesis to justify the difference between the accuracy of CROP index and that of IWI is that in the first one, the MIP (a criterion that is considered to be less accurate) [11,21] was included, and in IWI, f/Vt ratio (an index that is considered to be one of the most accurate) [11,16] was included. We think that MIP inclusion in CROP index impairs its accuracy.

In a prospective study by Aboussouan and colleagues [12] the time to weaning evaluated in 113 consecutive patients was shorter in those that presented the Cst,rs of more than 20 ml/cmH_2_O, a normal creatinine level (0.6 to 1.4 mg/dl) and a f/Vt ratio of 105 breaths/min/liter or less. Our results corroborated the findings of the study by Aboussouan and colleagues [12], once Cst,rs and f/Vt ratio are included in the IWI equation and presented the second and the third largest areas under the ROC curves (0.85 and 0.83, respectively).

Our results showed that the integration of important single functions into an index such as IWI can be helpful to improve its weaning predictive value when compared with each single function component alone. Patients that present poor prognosis for weaning according to a high f/Vt ratio (e.g. 120 breaths/minute/liter), can present good prognosis according to IWI, if Cst,rs and the SaO_2 _are higher than 35 ml/cmH_2_O and 90%, respectively. On the other hand, patients with a SaO_2 _less than 92% and a Cst,rs of 25 ml/cmH_2_O or less, even with a f/Vt ratio of 93 breaths/minute/liter, will present poor prognosis for weaning according to the IWI. So, the three components are essential for the accuracy of IWI and the fact that any of the three parameters is not favorable for weaning does not mean that IWI is not going to be favorable, either.

Regarding the evaluation of oxygenation by the IWI index, we preferred SaO_2 _to PaO_2_/FiO_2 _because SaO_2 _has fewer variations (generally higher than 90 to 92%) [1,2] than PaO_2_/FiO_2 _(higher than 150 to 200) [8,24-26] during the weaning of mechanical ventilation, being a better parameter to compose an accurate IWI. In the study by Khamiees and colleagues [25], most medically ill patients (89%) with PaO_2_/FiO_2 _ratios from 120 to 200 (four out five patients with PaO_2_/FiO_2 _ratios from 120 to 150), were extubated successfully. Krieger and colleagues [26] found that a PaO_2_/FiO_2 _ratio of 238 had a PPV of 90% and a NPV of only 10%.

### Main limitations of the study

Although Cst,rs can be measured during discontinuation from mechanical ventilation [11,12,27-29], it is not an easy task to be performed during the weaning process, because the patient's inspiratory effort during the assisted breath could interfere with the inspiratory plateau pressure measurement. In our study we minimized this limitation by observing the digital display of the pressure-time inspiratory plateau curve thus avoiding respiratory cycles that revealed clear inspiratory efforts of the patients.

In our study, the IWI was measured with a fixed FiO_2 _of 35% in order to avoid variations in SaO_2 _due to FiO_2 _variations. Further studies must be performed to test the IWI accuracy in a wide range of FiO_2 _values.

The measurement of the tracheal P 0.1 can be a limitation of the study because P 0.1 is traditionally measured through an esophageal balloon. However, tracheal P 0.1 can be accurately measured at the bedside [30,31] through a new generation of software coupled to microprocessor mechanical ventilators, thus being an easier form of P 0.1 assessment than the esophageal balloon technique.

## Conclusions

The use of an index, such as IWI, that integrates important weaning parameters can evaluate the weaning outcome with better accuracy. A satisfactory oxygenation and Cst,rs when associated with an adequate breathing pattern, generally leads to a successful weaning. The opposite generally leads to an unsuccessful weaning. In our study the comparison of IWI to other traditional weaning indexes revealed that IWI was the best index to predict the weaning outcome.

## Key messages

• The f/Vt ratio remains one of the best predictors of weaning outcome.

• IWI is an index that comprises respiratory system compliance, which informs about the mechanical condition of the lungs and chest wall; SaO_2_, which provides information about the patients' capacity to maintain a desirable oxygenation and f/Vt ratio, which informs about the patients' capacity to maintain unassisted breathing, evaluating the weaning outcome with better accuracy.

• IWI was useful to detect those patients who passed the SBT but needed reintubation afterwards.

• In our population, IWI was the best index to predict the weaning outcome.

## Abbreviations

COPD: chronic obstructive pulmonary disease; CROP: acronym of compliance, rate, oxygenation and pressure; Cst,rs: static compliance of the respiratory system; DA: diagnostic accuracy; FiO_2_: fraction of inspired oxygen; f: respiratory rate; f/Vt ratio: frequency to tidal volume ratio; ICU: intensive care unit; IWI: integrative weaning index; LR+: likelihood ratio of positive test; LR-: likelihood ratio of negative test; MIP: maximal inspiratory pressure; NPV: negative predictive value; P 0.1: airway occlusion pressure; PaCO_2_: partial pressure of arterial carbon dioxide; PaO_2_/FiO_2 _ratio: ratio of arterial oxygen tension to fraction of inspired oxygen; PEEP: positive end expiratory pressure; PPV: positive predictive value; P(W+/T+): probability for weaning success if test is positive; P(W+/T-): probability for weaning success if test is negative; ROC: receiver operator curve; RSBI: rapid shallow breathing index; SaO_2_: arterial oxygen saturation; SBT: spontaneous breathing trial; SE: sensitivity; SP: specificity; Vt: tidal volume.

## Competing interests

The authors declare that they have no competing interests.

## Authors' contributions

All authors, except RN, equally contributed to the design, data acquisition and manuscript preparation. RN (from the Biostatistics Department of Federal University of Rio d Janeiro - Rio de Janeiro - Brazil) wrote the statistical analysis.
